# Physicochemical Characterization of Cherry Pits-Derived Biochar

**DOI:** 10.3390/ma15020408

**Published:** 2022-01-06

**Authors:** Vladimír Frišták, Diana Bošanská, Martin Pipíška, Libor Ďuriška, Stephen M. Bell, Gerhard Soja

**Affiliations:** 1 Department of Chemistry, Trnava University in Trnava, 91843 Trnava, Slovakia; diana.bosanska@tvu.sk (D.B.); martin.pipiska@truni.sk (M.P.); 2 Institute of Materials Science, Faculty of Materials Science and Technology in Trnava, Slovak University of Technology in Bratislava, 91724 Trnava, Slovakia; libor.duriska@stuba.sk; 3 Institute of Environmental Science and Technology (ICTA-UAB), Universitat Autónoma de Barcelona, 08193 Barcelona, Spain; Stephen.Bell@uab.cat; 4 Energy Department, Austrian Institute of Technology GmbH, 3430 Tulln, Austria; Gerhard.Soja@ait.ac.at; 5 Institute for Chemical and Energy Engineering, University of Natural Resources and Life Sciences Vienna, 1190 Vienna, Austria

**Keywords:** biochar, pyrolysis, sorption separation, Hg, As

## Abstract

Although the suitability of some biochars for contaminants’ sorption separation has been established, not all potential feedstocks have been explored and characterized. Here, we physicochemically characterized cherry pit biochar (CPB) pyrolyzed from cherry pit biomass (CP) at 500 °C, and we assessed their As and Hg sorption efficiencies in aqueous solutions in comparison to activated carbon (AC). The basic physicochemical and material characterization of the studied adsorbents was carried out using pH, electrical conductivity (EC), cation exchange capacity (CEC), concentration of surface functional groups (Boehm titration), and surface area (SA) analysis; elemental C, H, N analysis; and Fourier-transform infrared spectroscopy (FTIR) and scanning electron microscopy with energy-dispersive X-ray spectroscopy (SEM–EDX). AsO_4_^3−^ anions and Hg^2+^ cations were selected as model contaminants used to test the sorption properties of the sorption materials. Characterization analyses confirmed a ninefold increase in SA in the case of CPB. The total C concentration increased by 26%, while decreases in the total H and N concentrations were observed. The values of carbonate and ash contents decreased by about half due to pyrolysis processes. The concentrations of surface functional groups of the analyzed biochar obtained by Boehm titration confirmed a decrease in carboxyl and lactone groups, while an increase in phenolic functional groups was observed. Changes in the morphology and surface functionality of the pyrolyzed material were confirmed by SEM–EDX and FTIR analyses. In sorption experiments, we found that the CPB showed better results in the sorption separation of Hg^2+^ than in the sorption separation of AsO_4_^3−^. The sorption efficiency for the model cation increased in the order CP < CPB < AC and, for the model anion, it increased in the order CPB < CP < AC.

## 1. Introduction

Aqueous organic or inorganic xenobiotics pose several threats to human society, leading to hazardous health issues such as respiratory allergies, skin diseases, gastrointestinal complications, infertility, and cancer [1]. Organic pollutants can be metabolized, degraded, or volatilized, but inorganic pollutants such as heavy metals are redispersed in different parts of the environment [2]. Although heavy metal ions occur naturally in the environment, their concentrations are now often elevated due to the intensification of industrial activities. Toxic elements enter the soil system through water bodies and subsequently infiltrate the food chain and, ultimately, human bodies [3,4]. As waste and wastewaters are the main source of heavy metals, they need to be treated to immobilize pollutants and/or convert them into less toxic forms. A large part of research is currently devoted to the development of efficient treatment technologies based on physicochemical, electrochemical, or oxidative processes. Physicochemical processes include membrane filtration, chemical precipitation, ion exchange, and adsorption.

Commercially available activated carbon as a sorption material has received much of the interest in the field of wastewater treatment and purification due to its valuable characteristics and properties. Thermal stability, homogenous surface morphology, low toxicity hazard, and high porosity and, thus, specific surface area of activated carbon predetermine its various applications in technological processes [5]. However, the cost of activated carbon production is relatively high, mainly due to post-production activation processes by chemical and physical methods [6]. The thermochemical conversion of feedstocks into new and innovative value-added products can be accomplished by several processes such as torrefaction, pyrolysis, hydrothermal carbonization, combustion, and gasification [7]. European Directive 2008/98/EC and Regulation 2019/1009 consider pyrolysis and gasification as recovery operations by the re-conversion of organic wastes [8,9]. Biochar, as the main product of thermochemical conversion techniques, is a carbon- and nutrient-rich material that is suitable for agriculture and the environment, and especially safe for humans, animals, and plants. Its increased porosity and surface area, as a result of pyrolysis conversion, allow its use as a soil fertilizer, immobilization agent, and potential new sorption material for the urgent removal of pollutants from contaminated aqueous solutions and liquid wastes [10]. A variety of feedstock materials can be used to produce biochar under strict anoxic conditions at pyrolysis temperatures of 350–700 °C. The wide range of potential feedstocks includes vegetable waste, fibrous vegetable waste from virgin pulp production, processing residues, biowaste, plant residues, and dead organisms or their parts [10,11,12,13,14,15]. Therefore, in recent decades, there has been a tremendous scientific boom in the field of biochar applications. The main focus of research has been on the optimization of process parameters such as pyrolysis temperature, residence time, and feedstock origin. In general, the transformation of biomass waste into biochar and activated carbon opens up several opportunities: (1) creating a revenue stream from otherwise discarded waste; (2) increasing nutrient use efficiency and soil stability; (3) mitigating drinking water pollution; (4) reducing anthropogenic environmental impacts of industrial food production. In the context of Central Europe, this biomass conversion would use waste from local sources to mediate local problems, thereby reducing the long-distance transport of wastes and byproducts to improve the environment and health of the local population.

Total fresh cherry production in recent years in the EU was about 794,000 metric tons/year [16]. The consumption and processing of this fruit by the food industry produces waste in the form of cherry pits that varies in quantity from state to state. For instance, the amount of cherry pits produced in Slovakia based on statistical data was assumed to be 76 tons per year as a result of fruit consumption alone [17]. Additionally, significant quantities of cherry pits also come from other areas of the food and distillery industries. In recent years, there have been several studies on the recovery of sour cherry pits [18,19,20]. The oils obtained from cherry pits are recommended for use in cosmetics and cooking due to the presence of fatty acids [19]. Studies have demonstrated the potential of pyrolysis of cherry pit waste for recovery as a renewable fuel, and others for co-firing biomass with coal to generate electricity. Other studies have suggested the use of cherry pit bio-waste as catalyst supports, electrode materials, adsorbents of alkaline gases, and as soil amendments for greenhouse crops improvement [20,21,22,23]. Kahraman and Pehlivan [24] studied biochar produced from cherry pits (*Prunus avium)* as a sorption material for Cr^6+^ ions removal from aqueous solutions. The pyrolysis process of cherry seed in the presence of sodium carbonate has been optimized by Balat [25]. The potential of cherry pits-derived biochar as a tool in nutrient cycles closing was described in the work by Barber et al. [26]. The authors studied the pyrolysis product in filtration experiments with its subsequent application as a soil fertilizer. Diacon et al. [6] showed the potential utilization of chemically activated cherry pits-derived biochar as carbon-based photocatalysts impregnated with different zinc salt precursors.

Unfortunately, despite the potential advantages, there have been limited studies conducted on the recovery of cherry pits for use as activated carbon (specifically, a highly porous material with a wide applicability in gas and liquid purification processes and in catalysis by a two-step physical activation method) [27]. In the whole of Central Europe, while cherry pits are abundantly found as a residual product of industrial cherry production, industry and waste management utilize cherry pits very rarely. In light of this situation and the need for more information to guide future research and industrial practices, this study focused on the preparation and characterization of cherry pits-derived biochar as a starting material for activated carbon through chemical or physical activation. Prepared pyrolysis was tested in a preliminary batch sorption test with As and Hg as model pollutants present in aqueous systems in anionic and cationic forms. Mercury as one of the most hazardous and toxic elements can cause serious damage to animal and human nervous systems. Arsenic has been recognized as a highly poisonous metalloid for flora and fauna and has been classified as carcinogenic for humans.

## 2. Materials and Methods

### 2.1. Feedstock Origin and Biochar Pyrolysis

Cherry pits (CPs) biomass (Natur-Haus-Kaiser, Neunkirchen, Germany) was washed in deionized water and oven-dried (60 °C). To attain a representative sample, 5 kg of prepared feedstock was homogenized. Noncrushed CPs were pyrolyzed in a continual pyrolysis reactor Pyreka 2.1 (Pyreg, Dörth, Germany) at 500 °C, with 20 min of residence time. To ensure anoxic conditions, N_2_ was used as a flushing gas (2 L/min). The obtained product, cherry pit biochar (CPB), was homogenized, crushed, and sieved to obtain the fraction of particles between 250 and 1000 µm. For comparison, the nonpyrolyzed biomass of CPs and commercially available activated carbon (AC) were homogenized and sieved to obtain the same particle size. All the obtained materials were stored in polypropylene tubes and used in characterization experiments.

### 2.2. Physicochemical Characterization

*pH and EC determination*

The pH values of CP, CPB, and AC were determined in deionized water (pH 7.10) and 1 mol/L KCl (pH 6.95) (*v*/*v* 1/10) by a pH multimeter after 1 h of shaking (150 rpm) and 1 h of stabilization. Electrical conductivity (EC) was determined in biochar suspension with deionized water (*v*/*v* 1/7.5) after 24 h of shaking at 45 rpm and 22 °C.

*CEC determination*

Cation exchange capacities (CEC) of CP, CPB, and AC were determined with the application of BaCl_2_ as an extraction agent according to the ISO 11260 method modified by Frišták et al. [2] for biochar samples. Specifically, 6 mL of 0.1 mol/L BaCl_2_ solution was added to 0.5 g of CP, CPB, and AC each. The samples were stirred for 1 h on a laboratory shaker at 45 rpm and 22 °C. After an hour, the supernatant was separated from the sedimented sample by centrifugation for 5 min at 4000 rpm. This step was repeated twice. In the next step, 3 mL of 0.025 mol/L BaCl_2_ solution was added to the sample in the tubes and stirred on a laboratory shaker for the next 19 h at 45 rpm and 22 °C. After the desired contact time expired, the supernatant was separated from the sediment sample by centrifugation (5 min, 4000 rpm). Afterward, 3 mL of 0.02 mol/L MgSO_4_ solution was added to the sample and the suspensions of CP, CPB, and AC were stirred for 19 h at 45 rpm and 22 °C. The supernatant separated by centrifugation (5 min, 4000 rpm) was used in the chelatometric determination of Mg cations using a standard solution of 0.02 mol/L Na_2_EDTA. The value of cation exchange capacity was calculated using Equation (1):

CEC = (M_0_ · V_0_ − M · V_V_) · ε · 10^3^ (1)

where M_0_ represents the molar concentration of Mg added to the sample (mol/L), V_0_ is the volume of Mg solution added to the sample (L), M represents the concentration of Mg in the titrated sample (mol/L), and V_V_ is the volume of the titrated sample (L).

*Total carbonates content determination*

For the determination of carbonates as an inorganic form of carbon in CP, CPB, and AC samples, the volumetric method using a laboratory Janko’s calcimeter was used. Janko’s calcimeter is based on the measurement of the volume of CO_2_ released during the decomposition of carbonates by HCl according to Equation (2):

CaCO_3_ + MgCO_3_ + 4 HCl → CaCl_2_ + MgCl_2_ + 2 H_2_O + 2 CO_2_ (2)

After the calcimeter preparation (filling the tube system with water to zero, closing the three-way valve), 5 g of CP, CPB, or AC sample was added into the decomposition glass flask. The smaller storage flask was filled with HCl (1:1). The reservoir flask with the rubber stopper was inserted into the digestion flask and the valve was turned to such a position that the digestion flask and the calibration tube were connected. By tilting the flask, HCl was poured onto the tested sample. The released CO_2_ passed through the hose into the first tube, from which it displaced water into the second (open) tube. The method was calibrated by CaCO_3_ standard application (Sigma Aldrich, Taufkirchen, Germany)

*Ash content*

The method according Rehrah et al. [28] to determine the amount of ash in samples of CP, CPB, and AC was used. After weighing 0.25 g of CP, CPB, and AC into cleaned and perfectly dried annealing ceramic crucibles, the samples were put in a laboratory muffle furnace at 700 °C. After 60 min, the samples were transferred into a laboratory desiccator, cooled to laboratory room temperature, and weighed. The ash content was calculated according to Equation (3):

ash (%) = (m_700_/m_0_) · 100 (3)

where m_700_ is the mass of the sample after combustion at 700 °C in a muffle furnace, and m_0_ is the original mass of the sample.

*Elemental and surface area analysis*

The total C, H, and N contents in CP, CPB, and AC were determined by elemental analysis (CHN-S Elemental Analyzer, EA 1108, Carlo Erba, AIT, Vienna, Austria). Total As and Hg contents were quantified by CV-AFS and ICP-MS (Perkin Elmer, Elan DRCe 9000, AIT, Vienna, Austria) after prior digestion of the samples using the HNO_3_/H_2_O_2_ method [29]. Surface area (SA) was analyzed by the gaseous N_2_ adsorption method (SORPTOMATIC 1990, AIT, Vienna, Austria) using the Brunauer–Emmett–Teller (BET) adsorption isotherm for data evaluation.

*Surface functional groups (Boehm titration)*

For the determination of surface functional groups, the method according to Goertzen et al. [30] was used. In plastic tubes (25 mL), 12.5 mL of 0.05 mol/L NaOH, NaHCO3, Na_2_CO_3_, and HCl was added to 0.25 g of CP, CPB, and AC samples. The tubes were sealed and stirred on a laboratory orbital shaker (Orbital shaker Multi-RS 60, Biosan, Latvia) for 48 h at 45 rpm and 22 °C. At the end of the stirring period, 10 mL from the resulting suspension was removed and transferred to new tubes to which 30, 20, and 30 mL of 0.05 mol/L HCl were added (the addition of the acid matched the above solutions at the beginning of the experiment, with no solution added to the supernatant with HCl). The solutions were stripped of excess CO_2_ by bubbling with N_2_ gas for 25 min before titration. After addition of the indicator (methyl red), the sample was titrated with a 0.05 mol/L NaOH solution until the yellow color transition. All measurements were performed in triplicate. The resulting concentrations of carboxyl, lactone, phenolic, and total base surface functional groups were calculated by the following mathematical relationships, following Equations (4)–(6):

(4) $$n\left(\mathrm{HCl}\right)=\frac{V_{0}\left(V_{a}\cdot C_{0}-V_{t\left(\mathrm{NaOH}\right)}\cdot C_{t\left(\mathrm{NaOH}\right)}\right)}{m\cdot V_{a}}$$

(5) $$n\left(\mathrm{NaOH}/{\mathrm{NaHCO}}_{3}\right)=\frac{V_{0}\left[(V_{a}\cdot C_{0}-\left(V_{\mathrm{HCl}}\cdot C_{\mathrm{HCl}}-V_{t\left(\mathrm{NaOH}\right)}\cdot C_{t\left(\mathrm{NaOH}\right)}\right)\right]}{m\cdot V_{a}}$$

(6) $$n\left({\mathrm{Na}}_{2}{\mathrm{CO}}_{3}\right)=\frac{2V_{0}\left[(V_{a}\cdot C_{0}-\left(\frac{V_{\mathrm{HCl}}\cdot C_{\mathrm{HCl}}-V_{t\left(\mathrm{NaOH}\right)}\cdot C_{t\left(\mathrm{NaOH}\right)}}{2}\right)\right]}{m\cdot V_{a}}$$

where n represents the concentration of functional groups on the surface of the CP, CPB, and AC (mmol/g), V_0_ is the volume of the initial NaOH/NaHCO_3_/Na_2_CO_3_/HCl solution (mL), C_0_ represents the concentration of the solutions with volume V_0_ (mol/L), V_a_ is the volume of the solution taken after 48 h of stirring (mL), and V_HCl_ and C_HCl_ are the volume (mL) and the concentration (mol/L) of the titrant (NaOH solution), respectively.

The NaOH solution was used to neutralize carboxyl, phenolic, and lactone functional groups. The concentration of carboxyl functional groups was calculated as the difference of n(NaOH) and n(NaHCO_3_), or n(NaOH) and n(Na_2_CO_3_), which results in the concentration of carboxyl and lactone functional groups.

*FTIR analysis*

Spectral analysis in the infrared region was used to determine the abundance of each functional group on the CP and CPB surface. AC analysis was not feasible, due to the nature of the sample itself. Prior to analysis, representative fractions of CP and CPB samples (0.25–0.1 mm size fraction) were dried at 60 °C for 24 h to remove excess moisture. FTIR analysis was performed using an iS50 FT-IR spectrometer in conjunction with an ATR module, measuring 32 scans, at a resolution of 4 cm^−1^ in the wavenumber range 4000–450 cm^−1^.

*SEM–EDX analysis*

The surface morphology of CP, CPB, and AC samples was monitored using scanning electron microscopy (SEM) as well as electron-dispersive X-ray analysis (EDX) to map the abundance of individual elements on the surfaces of the materials. The analysis was carried out using a JEOL JSM 7600F electron microscope (Akishima City, Tokyo, Japan) at 20 kV, a vacuum pressure of 9.0 × 10^−4^ Pa, and magnifications of 250× and 1500×.

### 2.3. Batch Sorption Test

The sorption potential of the CP, CPB, and AC was verified in model experiments using selected Hg^2+^ and AsO_4_^3−^ sorbates. The sorption experiments were carried out in a batch experimental design according to OECD Methodology No. 106 [31]. In short, 0.25 g of CP, CPB, and AC was added into plastic (for As) and glass (for Hg) tubes, and we pipetted 7.5 mL of AsO_4_^3−^ solution with a concentration of 0.2 mmol/L and Hg^2+^ solution with a concentration of 0.5 mmol/L into these tubes. Stock solutions were prepared from HgCl_2_ and Na_2_HAsO_4_. The pH value of both solutions was in the range of 5.5–6.0. The samples were stirred on a laboratory orbital shaker (Orbital shaker Multi-RS 60, Biosan, Latvia) at 45 rpm, for 24 h at 22 °C. After the contact time, the supernatant was separated from the sedimented sample by centrifugation and filtrated through a 0.45 μm nylon filter. The concentration of Hg and As sorbed by CP, CPB, and AC-based adsorbents was determined from the Hg and As present in the supernatant. The quantification of both elements was carried out using an EcaFlow electrochemical analyzer (ISTRAN, Slovakia). All measurements were performed in triplicate. The basic relationship below, Equation (7), was used to calculate the sorption efficiency of Hg and As adsorbents based on CP, CPB, and AC with a fraction size of 0.25–0.1 mm:

(7) $$\eta \;\left(\%\right)=\frac{\left(c_{0}-c_{m}\right)}{c_{0}}\cdot 100$$

where η represents the adsorption efficiency (%), c_0_ is the concentration of Hg or As before the sorption experiment (at time t_0_) (mg/L), and c_m_ is the concentration of Hg or As after the sorption process (at time t = 24 h) (mg/L).

### 2.4. Electrochemical Analysis of Hg and As

**Figure 1:**
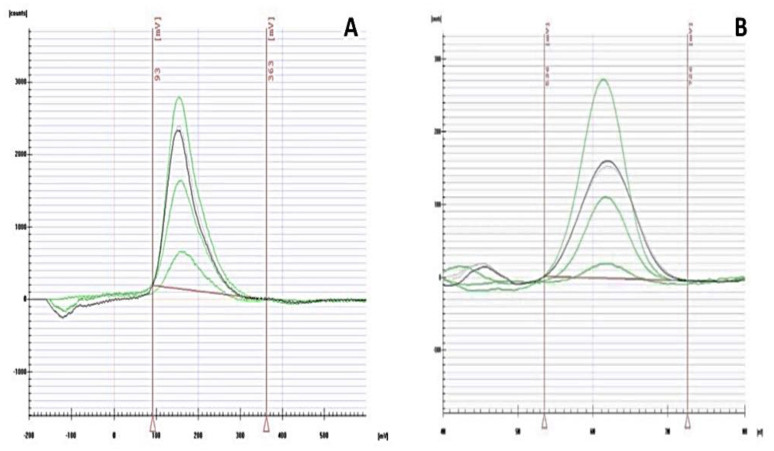
Characteristic chronopotentiogram for the determination of As (**A**) and Hg (**B**) in supernatant samples after sorption by CP, CPB, and AC-based adsorbents.

The supernatant samples obtained after the sorption experiments were analyzed for free As and Hg content using an EcaFlow 150 electrochemical analyzer with an E-T/Au electrode, which operates on the principle of dissolution chronopotentiometry. For the actual determination, the application sheets and the manufacturer’s recommended methodology for the determination of total As in water samples (Application Sheet No. 41, ISTRAN, Slovakia) and low concentrations of Hg in water samples (Application Sheet No. 51, ISTRAN, Slovakia) were applied (Figure 1).

## 3. Results and Discussion

### 3.1. Material Characterization

The resulting pH values of the samples were in the alkaline range for commercial activated carbon and biochar prepared from cherry pits (Table 1). Due to the pyrolysis temperature (500 °C) used to prepare the biochar, it was expected that the pH values of the biochar would be >9. Jindo et al. [32] reported very similar pH values for biochar samples prepared from apple twigs, rice husk, oak, and rice straw, and as the pyrolysis temperature increased, the pH values also increased. This phenomenon likely takes place due to the relative concentration of unpyrolyzed inorganic elements present in the original biomass. The pH value of the cherry pit biomass was in the acidic region, which was also reflected when the concentrations of functional groups on the surface of the sorbents were detected by Boehm titration (Table 2). Cherry pit biomass showed higher concentrations of lactone and carboxyl functional groups, while an increase in phenolic surface functional groups was observed in CPB. The potential pH values were in the order CP < CPB < AC, toward the alkaline region.

The electrical conductivity of CP significantly exceeded the electrical conductivity values of CPB and AC. This difference was more than 10 times higher than the conductivity values of CPB and AC. The EC values depend on the feedstock and pyrolysis temperature. Kloss et al. [10] observed similar electrical conductivity values for biochar prepared from straw and woodchip-based biochar. The biochar prepared from straw showed higher EC values.

The values obtained for the ash content of the CP, CPB, and AC showed that cherry pits biomass contained the highest percentage of ash. In this case, it was assumed that the biochar prepared by the pyrolysis of cherry pits would show the highest value. Duran-Valle et al. [23], who investigated the same type of biomass at different pyrolysis temperatures, reported that the volatility of the substances contained in the CP increases with pyrolysis temperature or time, which is reflected by a decrease in the volatile content of the resulting carbonized product, an increase in the ash content, and a constant carbon content. This is a consequence of the concentration of the inorganic fraction and carbon in such a product. In our case, it is possible that the initial pre-treatment of the material, namely washing with deionized water, removed the fractions responsible for the excessive production of ash, or that the method used did not result in the complete ashing of the CPB sample.

**Figure 2:**
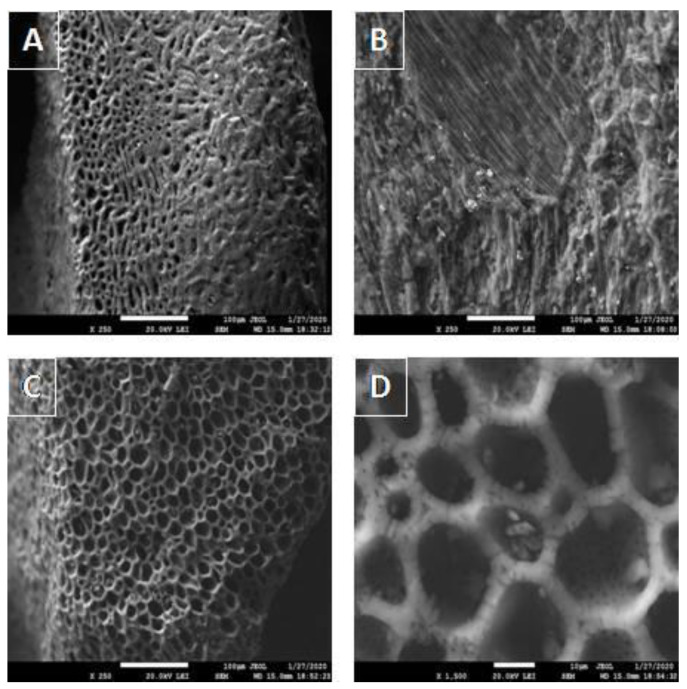
SEM images of CP (**A**), AC (**B**), and CPB (**C**,**D**) at magnifications of 250× and 1500×.

After analyzing the cation exchange capacity, we found that CPB was able to sorb half as many cations as the primary biomass (CP) could, confirming its potential in sorption separations. However, compared to AC, the value of cation exchange capacity was half as high. The surface size of CPB increased approximately ninefold compared to that of CP due to the formation of macro-, meso-, and micropores. Duran-Valle et al. [23] also concluded that the effect of pyrolysis temperature and residence time on biochar porosity is not very significant. Approximately half of the porosity is composed of macropores. Such a small volume of mesopores is indeed surprising because mesopores are the pores that guide adsorbing molecules to the micropores, and these pores are where the surface area of a given porous solid is largely concentrated. The micropore openings are located in the outer surface and in the walls of the macropores (Figure 2D). Different types of biomass exhibit different surface sizes at a pyrolysis temperature of 500 °C, e.g., in the case of pine needle-based biochar, the surface size is 236 m^2^/g [33].

Elemental analysis of the samples showed that there was an increase in total carbon in CPB compared to CP, while the H content decreased after pyrolysis treatments. We also observed a significant decrease in total N in CPB. Frišták et al. [2] stated that the degree of carbonization of biomass can be demonstrated by the level of hydrogen, which is primarily associated with the organic matter of the plant feedstock. In comparison, the biochar prepared from beech wood chips analyzed in our previous work contained less H and N, and conversely more C.

**Figure 3:**
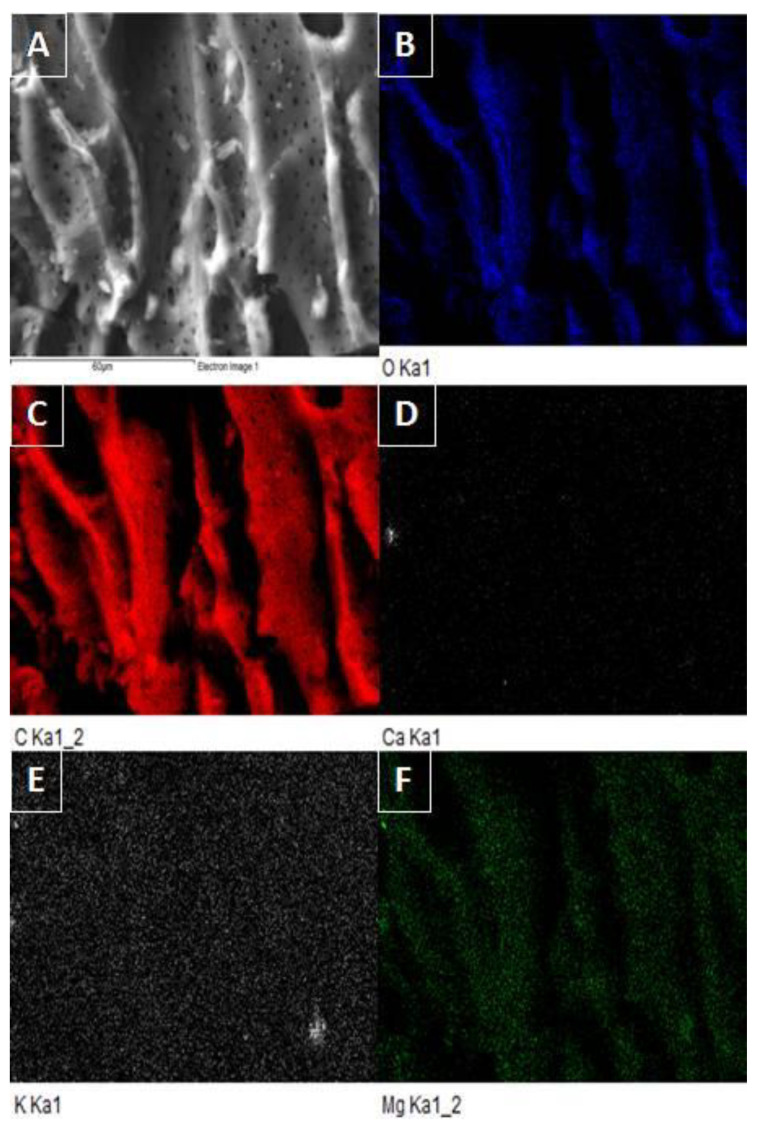
SEM image of CPB (**A**) and EDX mapping of selected area of CPB for O (**B**), C (**C**), Ca (**D**), K (**E**), and Mg (**F**).

Scanning electron microscopy was used to closely analyze the surface of CP, CPB, and AC samples. The obtained micrographs of CPB (Figure 2) were taken at 250× and 1500× magnification. From the comparison of the CP (Figure 2A) and CPB (Figure 2C) images, it is evident that there was a significant change in the porosity of the pyrolysis material and the creation of new surface structural formations. Pyrolysis treatments of biomass increase the volumes of both internal and external pores, implying an increase in both internal and external surfaces capable of binding particles during sorption processes [2,4], which was also confirmed by surface size analysis (Table 1). Micrograph D represents a 1500-fold magnification of a CPB sample, in which micropores are clearly visible (as confirmed by Figure 2A), and micrograph B represents the comparable sample AC. The studied biochar sample was also subjected to energy-dispersive X-ray analysis (EDX) to map its surface in detail, focusing on C, O, Ca, K, and Mg, with C being the majority. Conversely, Ca showed the lowest abundance (Figure 3D). O and Mg are also relatively abundant (Figure 3B–F), indicating that oxides and oxohydroxides of Mg, K, and Ca are present on the surface.

**Figure 4:**
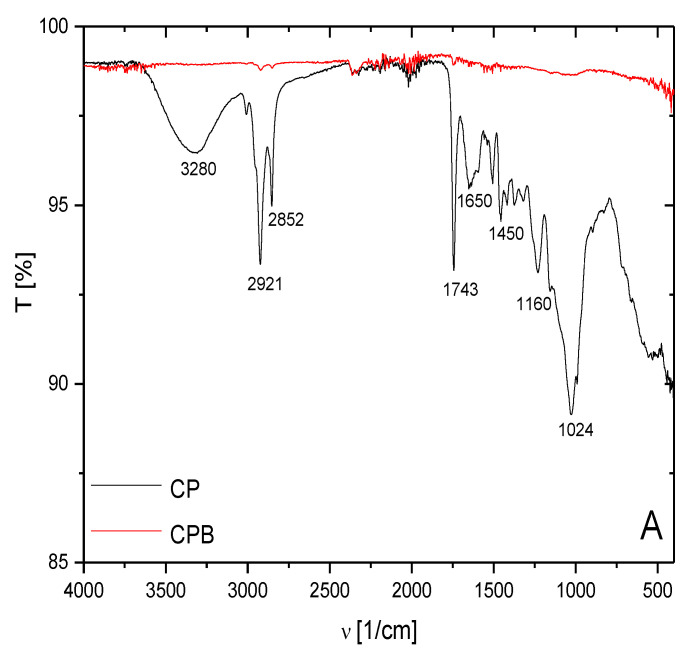

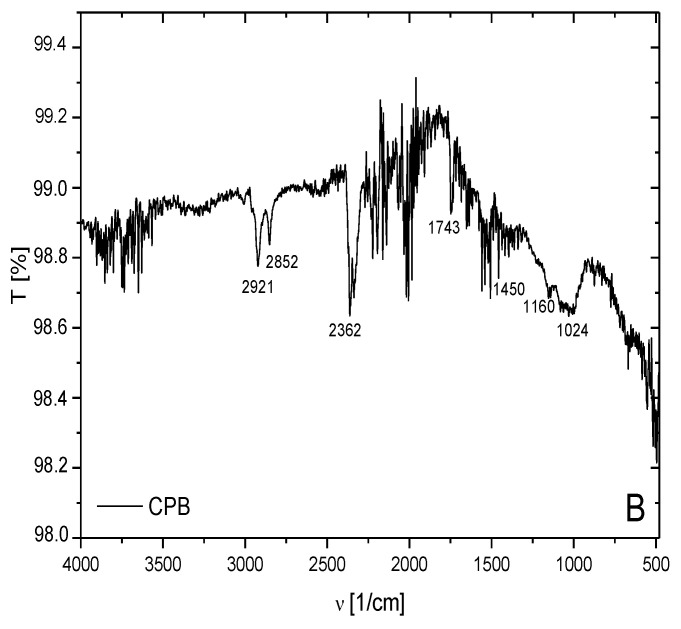
FTIR spectra of CP (**A**) and CPB (**A**,**B**) in the range of wavenumbers of 450–4000 cm^−1^. (**B**) Enlargement of CPB spectrum in the transmission range 98–99.5%.

Spectral analysis of CPB and CP samples in the IR region of the spectrum (Figure 4) showed changes in the absorption bands (peaks) of characteristic surface functional groups. In the case of CPB, a significant decrease also occurred in the spectral region at 1024 cm^−1^ (Figure 4A). Only the aromatic skeleton and ether-type structures are thermally stable when heated to high temperatures, which is evident in the 2390–2350 cm^−1^ region (Table 3). From the comparison of the IR spectra of CP and CPB samples, a decrease in the intensity of the absorption bands by the regions 3600–2300 cm^−1^ and 1800–400 cm^−1^ due to the influence of carbonization processes is evident. The aromatic structures present in CPB provide π-electrons, with the potential to strongly bind heavy metal cations. Asymmetric valence O-H vibrations, which are characteristic of the 3450–3050 cm^−1^ region, are typical for the presence of residual moisture in biomass [4,34]. A significant decrease was observed for aliphatic compounds represented by asymmetric C-H valence vibrations in the 2980–2820 cm^−1^ region and asymmetric C=O valence vibrations in the 1750–1700 cm^−1^ region. This wavenumber range is characteristic for vibrations representing carboxylic acids, ketones, and esters. The region of the spectrum from 1650 cm^−1^ to 1400 cm^−1^ corresponds to the asymmetric C=C vibrations of aromatics. In the mentioned spectral range, Molenda et al. [35] reported that spectral lines of biochar derived from corn waste at wavenumber 1617 cm^−1^ correspond to the vibration of the aromatic ring, and those at 1657 cm^−1^ correspond to the vibrational band of the C=O bond in quinone. In this work, in the case of biochar based on cherry pits, the absence of oxygen functional groups such as carbonyl or quinone, which occur independently on the biochar surfaces, is also mentioned. This fact was also confirmed by FTIR analysis of CPB. The assumption is that oxygen is incorporated into the aromatic carbon structures in the form of ether bridges, which can be demonstrated by the spectral bands present in the spectra of the products obtained at higher temperatures. Chen et al. [36] worked with biochar based on sewage sludge and, in their work, they described intensive absorption peaks at 3420 cm^−1^ and 1420 cm^−1^ that were observed at higher pyrolysis temperatures. At pyrolysis temperatures <500 °C, carbon products are formed that contain organic oxygen structural groups linked to carbon-carbon double bonds in aromatic rings. This is evidenced by the spectral bands located in the spectra of biochar samples obtained from wheat straw for wavenumber 1581 cm^−1^, from corn waste for wavenumber 1576 cm^−1^, and from cherry pit biochar for wavenumber 1585 cm^−1^ [34]. This analysis also demonstrated changes in the intensity of the peaks and density due to pyrolysis treatment of the biomass. Duran-Valle et al. [27] reported that the spectral lines at 3000, 1700, and 875 cm^−1^ become weaker or even absent in the case of cherry pits-derived biochar.

### 3.2. Model Sorption Test

**Figure 5:**
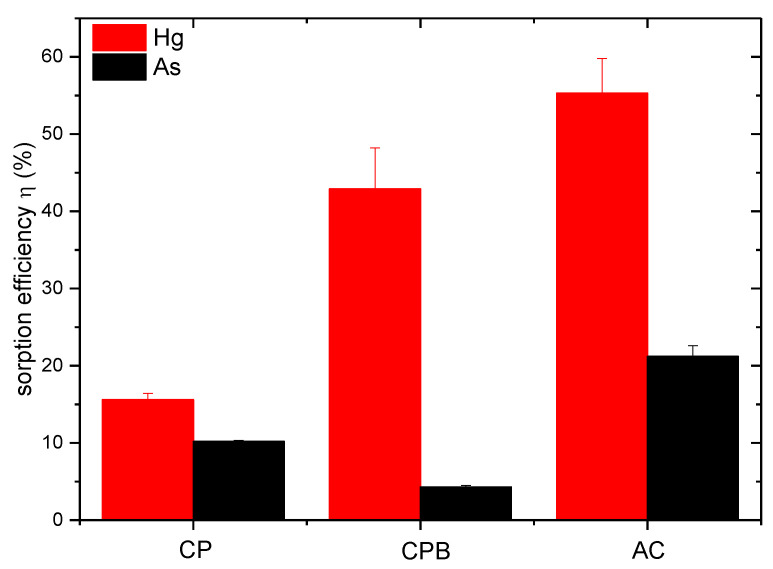
Efficiency of Hg and As sorption separation from aqueous solutions by CP, CPB, and AC under the following experimental conditions: sorbent to sorbate ratio 1:40, sorbent fraction 250–1000 μm, c_0_ (Hg) = 0.5 mmol/L, c_0_ (As) = 0.2 mmol/L; pH_0_ = 5.5–6.0; contact time 24 h at 22 ± 2 °C, stirring 45 rpm.

A model experiment in a batch sorption design was used to investigate the sorption properties of the analyzed biochar. We chose Hg and As as model contaminants, whose occurrence in water matrices increasingly exceeds concentration limits (Figure 5).

The sorption separation efficiency of CPB for the cationic form of Hg was higher than that of the anionic As, as expected from the preliminary CEC results (Table 1). The cherry pits-derived biochar showed very good results in the sorption of Hg, which was around 43%. In the case of CP, the sorption values found were much lower. From the data obtained for As sorption separation by the samples, it was the AC sample, which served as a comparative sorbent, that showed the best results. The lowest values of As sorption were for CPB. The sorption of Hg depends on the concentration of phenolic, hydroxyl, and carboxyl functional groups on sorption surfaces [37]. The CPB sample contained the highest abundance of phenolic compounds compared to the other samples. Li et al. [29] reported that pyrolysis temperature has a significant effect on As sorption. Biochar prepared at 600 °C had a lower sorption capacity than biochar prepared at 300 °C due to the loss of O-rich functional groups. These data confirm the importance of O-containing functional groups on the biochar surface for As sorption. Arsenic anions interact with positively charged functional groups by electrostatic attraction. At lower pH, biochar is positively charged with a higher degree of protonation of functional groups than high-pH groups [4]. Similar results were confirmed by Boni et al. [38] in a study focused on biochar application in As sorption separation in a column system. The active pH of CPB was alkaline in our case (Table 1). Surface chemical modification with Fe oxides could help increase the sorption potential of CPB for anionic forms of contaminants. Samsuri et al. [39] modified rice husk-based biochar with Fe oxides and observed more than a twofold increase in the sorption capacity of anionic As. Similarly, in the work of Micháleková-Richveisová et al. [4], the authors used chemical modification of corn cobs-derived biochar by Fe impregnation to increase the sorption capacity of the sorbent for PO_4_^3−^ removal from aqueous solutions. Subsequently, in our previous work, Frišták et al. [40], we used a similar modification of a biochar-based sorbent to improve the sorption potential of biochar for As removal from model solutions.

## 4. Conclusions

The subject of our investigation in this work was a biochar-based adsorbent, with the input biomass being cherry pits. By employing slow pyrolysis at 500 °C, we were able to prepare a pyrolysis product that was tested as a potentially useful material in sorption separations of contaminants from water, represented here by As and Hg because of their widespread occurrence. Material characterization, physicochemical properties as well as sorption experiments confirmed the applicability of the prepared material in wastewater treatment processes. The cherry pits-based CPB demonstrated considerable potential in the sorption separation of Hg from aqueous environments. The sorption efficiency increased in the order of CP < CPB < AC. The sorption efficiency of As was significantly lower for all tested sorbents, with the sorption success increasing in the order of CPB < CP < AC. Sorption rates were significantly enriched by the pH and concentration of surface functional groups. These findings warrant more detailed study, as well as further experiments devoted to the effects of reaction parameters (kinetic, initial concentration of sorbate, sorbent dose, pH) on sorption efficiency. However, the obtained results suggest the possible use of biochar-based adsorbents prepared from cherry pits in the sorption separations of cationic forms of contaminants. The sorption of anionic chemical forms of sorbates could be improved by biochar surface modification by physical or chemical methods.

## Figures and Tables

**Table 1:** Physicochemical properties of CP, CPB, and AC.

	CP	CPB	AC
pH_H2O_	6.13 ± 0.25	8.88 ± 0.36	8.54 ± 0.26
pH_KCl_	5.71 ± 0.32	6.69 ± 0.20	6.74 ± 0.27
EC (μS/cm)	248.55 ± 15.92	19.51 ± 1.40	19.45 ± 1.51
Ash content (%)	2.12 ± 0.11	1.61 ± 0.08	0.95 ± 0.20
CO_3_ (%)	7.9 ± 0.2	2.4 ± 0.1	ND ^2^
CEC (meq/100 g)	12.06 ± 0.53	28.21± 0.59	45.87 ± 0.98
SA (m^2^/g)	10.31	94.12	852
C (%)	47.74	74.41	92.81
H (%)	9.45	2.14	0.11
N (%)	12.14	4.87	0.88
As (mg/kg)	<LOD ^1^	<LOD ^1^	<LOD ^1^
Hg (mg/kg)	<LOD ^1^	<LOD ^1^	<LOD ^1^

^1^ value lower than limit of detection (LOD) for selected analysis (As 3 μg/L, Hg 5 μg/L); ^2^ value not detected.

**Table 2:** Concentrations of surface functional groups of CP, CPB, and AC determined by Boehm titration.

	Phenolic Groups (mmol/g)	Carboxylic Groups (mmol/g)	Lactonic Groups (mmol/g)	Total Basic Groups (mmol/g)
CP	0.29 ± 0.01	0.42 ± 0.03	0.39 ± 0.01	ND ^1^
CPB	0.68 ± 0.04	0.20 ± 0.01	0.11 ± 0.01	1.02 ± 0.02
AC	0.21 ± 0.02	ND ^1^	0.23 ± 0.05	0.98 ± 0.05

^1^ value not detected.

**Table 3:** Vibration wavenumber ranges of characteristic functional groups identified in CP and CPB (adapted from [27] and [2]).

FTIR Peak Position ν (1/cm)	Chemical Bond (Functional Group)
3450–3050	O–H stretching (Si-OH)
2980–2820	C–H stretching (alkyl CH)
2390–2350	O=C=O (carbonyl)
1750–1700	C=O stretching (ketones, carboxylic acids)
1650–1605	C=C stretching (aryl, double-bond)
1460–1400	aromatic C=C ring structure
1165–1114	aromatic C=O stretching
1060–1005	aromatic C=O stretching
